# Primary care as a setting for introducing milk using the milk ladder in children with IgE‐mediated cow's milk protein allergy

**DOI:** 10.1002/clt2.12286

**Published:** 2023-07-17

**Authors:** Caoimhe Cronin, Anne Marie McGinley, Laura Flores, Anne McKiernan, Roberto Velasco, Jonathan O’B Hourihane, Juan Trujillo

**Affiliations:** ^1^ Department of Paediatrics and Child Health University College Cork Cork Ireland; ^2^ The Weir Family Clinic Bandon Co Cork Ireland; ^3^ Department of Paediatrcs Cork University Hospital Cork Ireland; ^4^ Paediatric Emergency Unit Hospital Universitari Parc Tauli Barcelona Barcelona Spain; ^5^ Royal College of Surgeons of Ireland Dublin Ireland; ^6^ Cork University Hospital Irish Centre for Maternal and Child Health Research (INFANT) HRB Clinical Research Facility Cork (CRF‐C) Cork Ireland

To the editor

Cow's milk protein allergy (CMPA) is one of the most common food allergies in infancy and childhood.^1^ IgE‐mediated CMPA is managed in tertiary care (TC) centres in Ireland using the iMAP milk ladder.^2^ However, in primary care (PC) settings in Ireland, the use of the milk ladder is recommended only in the management of non‐IgE mediated CMPA.^3^

In a recent review, the milk ladder was shown to be a safe and effective method of gradual introduction to milk in children with IgE‐mediated CMPA.^4^ Two recent studies demonstrated the effectiveness of the iMAP milk ladder in tertiary allergy centres in Ireland and the UK^2^ ^,^ ^5^; however, the employment of the milk ladder in children with IgE‐mediated CMPA in non‐hospital clinical settings by general practitioners has not yet been evaluated. We reviewed the safety and effectiveness of the use of the milk ladder for children with IgE‐mediated CMPA in a PC setting.

This retrospective analysis is part of a larger study comparing the management strategies of IgE‐mediated CMPA in two countries (Ireland and Spain). Patients diagnosed with IgE‐mediated CMPA between 2015 and 2021 who were treated with the iMAP milk ladder in a tertiary paediatric allergy clinic and in a local PC clinic in Ireland were included. Parents were trained in the use of the milk ladder by clinical staff. An adapted Milk Allergy in Primary care (MAP) Guideline,^6^ known as the milk ladder was used. This ladder uses a 12‐step guideline for the reintroduction of foods containing different amounts of milk protein.

The main outcome was reaching step 12 on the iMAP milk ladder, the introduction of unrestricted liquid cow's milk, which was defined as the uneventful intake of more than 150 mL of cow's milk or the equivalent intake of 4.5 g of milk protein daily. Failure to complete the ladder was defined as the failure to introduce liquid cow's milk after 36 months of follow‐up. Data were analysed with SPSS Version 28.

A total of 13 patients in the primary care (PC) cohort and 69 patients in the TC cohort were included for analysis. Demographic features are shown in Table 1. Eleven (85%) patients in the PC cohort completed the reintroduction of milk to their diet compared to 57 (83%) in the TC cohort (*p* = 0.86). These results are similar to previous studies of the use of the milk ladder in IgE‐mediated CMPA.^2^ ^,^ ^5^

**TABLE 1:** Cohort demographics.

	Primary care (*n* = 13)	Tertiary care (*n* = 69)	*p* value
Male	6 (46.2%)	42 (60.9%)	0.323
Atopic dermatitis	10 (76.9%)	54 (78.3%)	0.915
Allergic rhinitis	0	6 (8.7%)	0.269
Egg allergy	7 (53.8%)	38 (55.1%)	0.935
Peanut allergy	1 (7.7%)	15 (21.7%)	0.241
Anaphylaxis before dx	1 (6.3%)	4 (5.8%)	0.793
Carries AAI	3 (23.1%)	9 (13%)	0.348
Age at diagnosis (months)	8.5 (4.8–12.31)	11.14 (9.2–13.04)	0.262
Mean (95% confidence interval (CI))			
SPT at diagnosis (mm)	5.6 (4.67–6.55)	4.7 (4.25–5.07)	0.064
Mean (95% CI)			
Total IgE at diagnosis (kU/L)	539 (*n* = 1)	558 (99.7–1017.86)	0.982
Mean (95% CI)			
IgE whole milk (kU/L)	51.4 (−58.94‐161.74)	21.74 (12.24–31.25)	0.161
Mean (95% CI)			

The mean time to completion of step 12 of the ladder was 12.7 months (95% CI 6.1–19.3 months) in the PC cohort and 15.5 months (95% CI 12.2–18.8 months) in the TC cohort (*p* = 0.472). Children in the PC group achieved tolerance at 25 months (95% CI 15.4–34.6 months) compared to 30.4 months (95% CI 24.87–35.98 months) in the TC cohort (*p* = 0.472).

Children in the PC cohort attended more appointments (mean 3.5 95% CI 1.3–5.6) than TC patients (mean 2.3 95% CI 2.0–2.7) (*p* = 0.047) but this did not affect rates of success in completing the milk ladder.

Allergic symptoms were experienced equally in the PC and TC groups (46.2% vs. 46.4% respectively, *p* = 1.0) with most presenting with either cutaneous symptoms (83.3% vs. 68.6%, *p* = 0.47) or gastrointestinal symptoms (33.3% vs. 40%, *p* = 0.76). No child suffered from anaphylaxis as a direct result of the managed introduction of milk using the milk ladder. Accidental exposure to milk during this time occurred in 3 (27.3%) patients in the PC cohort, and 8 (11.6%) patients in the TC cohort (*p* = 0.265). Two of these accidental exposures to milk resulted in anaphylaxis, one in each cohort. Mild allergic reactions occur commonly as the child escalates to a higher step on the ladder, and effective parent education regarding allergic reaction management allows for the child to continue to progress through the ladder.^5^ These results demonstrate that using the milk ladder in PC has safety outcomes similar to those in TC.

Strengths of this study include clinical staff that were identically trained in the same milk ladder guidelines in both settings, a sample size of patients from TC comparable to other published studies^2^ ^,^ ^5^ and the similar demographic characteristics of both cohorts. Limitations of this study include the small sample size of PC patients, and the retrospective nature of the data collection, resulting in a lack of clinical information found in some of the patients’ charts.

In conclusion, the results of this preliminary study suggest that PC is a safe and effective setting to employ the milk ladder as a method of reintroduction in children with IgE‐mediated CMPA. To our knowledge, this is the first study exploring the use of the milk ladder for IgE‐mediated CMPA in PC settings. Further prospective and randomized studies are recommended to support general practitioners in the use of the milk ladder in their clinical practice.

## AUTHOR CONTRIBUTIONS

Caoimhe Cronin: Data collection, statistical analysis, project management, writing the manuscript. Anne Marie McGinley: Data collection. Laura Flores: Creation of the clinical database, data collection, project management. Anne McKiernan: Data collection. Roberto Velasco: Supervision of data analysis. Jonathan O’B Hourihane: Supervision and editing of manuscript writing. Juan Trujillo: Management and supervision of the project, drafting and editing the manuscript. All authors have read and agreed to the published version of the manuscript.

## CONFLICT OF INTEREST STATEMENT

The authors declare no conflicts of interest.

## FUNDING INFORMATION

National Dairy Council and Dairy Research Ireland.

## CONSENT

This was a retrospective chart review involving the collection of anonymised patient data. Therefore, participant consent was not required.

## INSTITUTIONAL REVIEW BOARD STATEMENT

The study was conducted in accordance with the Declaration of Helsinki, and approved by the Clinical Research Ethics Committee of the Cork Teaching Hospitals ECM4(e) 13/4/2021.

## Data Availability

The data presented in this study are available only on request from the corresponding author due to restrictions, that is, privacy and ethics.
